# Infrared Spectroscopy of SARS‐CoV‐2 Viral Protein: from Receptor Binding Domain to Spike Protein

**DOI:** 10.1002/advs.202400823

**Published:** 2024-07-12

**Authors:** Tiziana Mancini, Salvatore Macis, Rosanna Mosetti, Nicole Luchetti, Velia Minicozzi, Andrea Notargiacomo, Marialilia Pea, Augusto Marcelli, Giancarlo Della Ventura, Stefano Lupi, Annalisa D'Arco

**Affiliations:** ^1^ Department of Physics University La Sapienza P.le A. Moro 2 Rome 00185 Italy; ^2^ Department of Basic and Applied Sciences for Engineering (SBAI) University La Sapienza Via A. Scarpa 16 Rome 00161 Italy; ^3^ Engineering Department University Campus Bio‐Medico of Rome Via Alvaro del Portillo 21 Rome 00128 Italy; ^4^ Centre for Life Nano‐ and Neuro‐Science Italian Institute of Technology Viale Regina Elena 291 Rome 00161 Italy; ^5^ Department of Physics University of Rome Tor Vergata Via della Ricerca Scientifica 1 Rome 00133 Italy; ^6^ Istituto di fotonica e nanotecnologie – Consiglio nazionale delle ricerche (CNR‐IFN) Rome 00133 Italy; ^7^ Laboratori Nazionali Frascati National Institute for Nuclear Physics (INFN‐LNF) Via E. Fermi 54 Frascati 00044 Italy; ^8^ RICMASS Rome International Center for Materials Science Superstripes Rome 00185 Italy; ^9^ Department of Science University Rome Tre Largo San Leonardo Murialdo 1 Rome 00146 Italy

**Keywords:** ATR‐IR spectroscopy, hydrophobicity, MultiFOLD, secondary structure, Spike glycoproteins

## Abstract

Spike (S) glycoprotein is the largest structural protein of SARS‐CoV‐2 virus and the main one involved in anchoring of the host receptor ACE2 through the receptor binding domain (RBD). S protein secondary structure is of great interest for shedding light on various aspects, from functionality to pathogenesis, finally to spectral fingerprint for the design of optical biosensors. In this paper, the secondary structure of SARS‐CoV‐2 S protein and its constituting components, namely RBD, S1 and S2 regions, are investigated at serological pH by measuring their amide I infrared absorption bands through Attenuated Total Reflection Infrared (ATR‐IR) spectroscopy. Experimental data in combination with MultiFOLD predictions, Define Secondary Structure of Proteins (DSSP) web server and Gravy value calculations, provide a comprehensive understanding of RBD, S1, S2, and S proteins in terms of their secondary structure content, conformational order, and interaction with the solvent.

## Graphical abstract

In the work “Infrared Spectroscopy of SARS‐CoV‐2 Viral Protein: from Receptor Binding Domain to Spike Protein” the Infrared spectral features of SARS‐CoV‐2 S protein and its constituting domains, namely RBD, S1, and S2 subunits, are investigated. Results are interpreted in terms of secondary structure content, intrinsic structural order and protein‐solvent interaction and compared with MultiFOLD models and DSSP assignment.

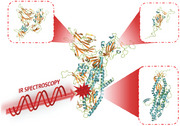

## 1 Introduction

The outbreak of COVID‐19, due to zoonotic virus SARS‐CoV‐2^[^ ^1^, ^2^, ^3^ ^]^ belonging to the β‐coronavirus (CoV) family, has raised many questions concerning the current public healthcare and surveillance systems. To mitigate and fight this pandemic, the scientific community is focused on techniques, approaches and practices for early monitoring the virus presence and to forbid the viral synthesis, for example by inhibiting the replicase enzyme or preventing the viral self‐assembly. In both cases, the main key target is the Spike (S) glycoprotein. The S protein of SARS‐CoV‐2 is a I‐th class fusion protein^[^ ^4^ ^]^ protruding from the surface of mature virions. It is the main responsible for receptor recognition and cell membrane fusion, being involved in the viral pathogenesis.^[^ ^5^, ^6^, ^7^ ^]^ Mature SARS‐CoV‐2 S protein has a size of ≈150 kDa and contains ≈1270 amino acids residues. It is composed of a signal peptide (≈13 aa) and two subunits, named S1 (≈600 aa) and S2 (≈540 aa). Figure S1 (Supporting Information) shows the structure of the SARS‐CoV‐2 S protein monomer (from Protein Data Bank ID:6vsb, by Wrapp et al.^[^ ^8^ ^]^) with its different domains clearly highlighted.

The S1 subunit contains a N‐terminal domain (NTD, ≈290 aa) and the receptor binding domain (RBD, ≈290 aa). The latter is the domain of the protein responsible for recognition and anchoring to the host receptor angiotensin‐converting enzyme 2 (ACE2)^[^ ^7^, ^9^ ^]^ triggering the endocytosis of the complex into the host cell. The S2 subunit contains the fusion peptide amino acids (FP, ≈30 aa) and the heptad repeat domains (including HR1, ≈70 aa), responsible for the membrane fusion.

Another structural feature of S proteins is their extensive glycosylation.^[^ ^10^, ^11^ ^]^ The CoV S proteins are densely covered with polysaccharide to camouflage and counteract the host immune response,^[^ ^10^, ^12^, ^13^, ^14^, ^15^ ^]^ participating in the S folding^[^ ^16^ ^]^ and working as recognition sites.^[^ ^17^ ^]^

The functionalities of these membrane proteins, such as cellular targeting and recognition, transport and communication^[^ ^18^, ^19^ ^]^ are affected by viral and host factors, such as the combination of immune evasion, the conformational masking of binding domains, and glycan shielding, as well as the extent of the receptor binding affinity and specificity.

In this context, the knowledge of the secondary structural characteristics of SARS‐CoV‐2 S protein and their components is of primary importance to understand the mechanisms occurring in the viral process and to address specific actions aimed at developing specific drugs, diagnostic tools and prevention actions.

Attenuated Total Reflection Infrared (ATR‐IR) spectroscopy^[^ ^20^, ^21^, ^22^ ^]^ is one of the well‐established experimental methods for a non‐invasive analysis of secondary structure of polypeptides and proteins.^[^ ^20^, ^21^, ^22^ ^]^ In particular, the amide I molecular vibration located between 1600 and 1710 cm^−1^ is the most sensitive to the protein secondary structure.^[^ ^20^ ^]^

On this basis, in a previous study^[^ ^23^ ^]^ we performed a comparative infrared vibrational spectroscopic study of the S1 glycoprotein monomers of MERS‐CoV, SARS‐CoV, and SARS‐CoV‐2, in aqueous solution at serological pH (7.4). The spectral component analysis of their amide I bands allowed us to reveal their complex secondary structure and observe significant differences among the IR features of these proteins, despite the high similarity in their amino acids sequences.^[^ ^23^ ^]^

In this work, we extend the study by addressing for the first time, at the best of our knowledge, the infrared features of the whole SARS‐CoV‐2 S glycoprotein monomer and its components, namely S1 and S2 subunits and the RBD domain, being these ones the main protein regions playing defined roles in the anchoring of ACE2 receptor. In this context, this study constitutes a first fundamental step in investigating the S protein. Further research will be devoted to the NTD, FP and HR1 components. In particular, we investigate the amide I vibrational band of the different S protein components, from the simple RBD up to the whole monomeric S protein, at serological pH (7.4), interpreting the results in terms of their secondary structure, hydrophobicity, and conformational order. Measuring the whole S protein and its S1, S2, and RBD components at serological pH provides a first fundamental step for further studies both in structural biology and biosensing fields. In particular for this last application, referring to S protein as a biomarker, its IR spectroscopic characterization first can provide a unique fingerprint allowing the optical detection of the virus. Moreover, the knowledge of its secondary structure obtained with a deep spectral analysis of amide I band, can provide further important information about the status and the activity/inactivity of the protein, being its secondary structure strongly related to its functionality.

Secondary structure contents estimated in terms of 𝛽‐sheet, random coil, 𝛼‐helix and 𝛽‐turn components for RBD, S1, S2, and S proteins are compared with DSSP assignments. Moving from the simpler RBD domain, to the more complex S1 and S2 subunits, up to the whole S protein, an evolution of IR spectral features is observed. IR spectral variations are interpreted in terms of secondary structure contents, intrinsic structural order and protein‐solvent interaction. All these results allow us to clarify the role of each S component and their contribution to the whole S protein conformational order and interaction. The knowledge of the secondary structure conformation of each domain and of their physico‐chemical features, contributes to shed light on S protein properties, its role and functionalities. Achieving S protein structure is preliminary for applicational studies on protein‐antibody interaction, viral infectious process, mutations conformational effects and protein dynamic in pathogenesis. Moreover, S protein secondary structure plays a main role in anchoring the ACE2 receptor. Therefore, knowing S protein three‐dimensional (3D) conformation at an interface paves the way for studies on S‐ACE2 interaction (and/or S‐antibody), on the dynamic of its structure during the interaction and to develop possible action for its inhibition or inactivation.^[^ ^24^, ^25^, ^26^ ^]^

## 2 Results

**Figure 1:**
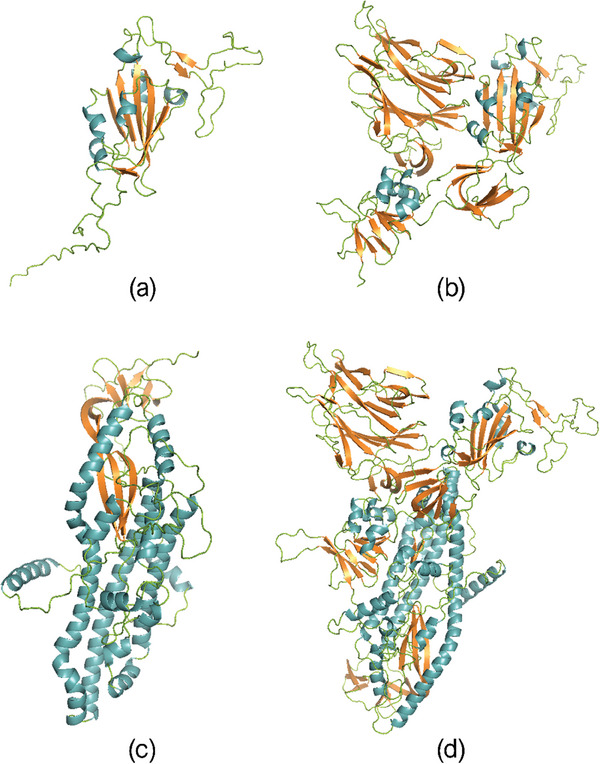
a) RBD region (319–541 aa), b) S1 subunit (1–685 aa), c) S2 subunit (686–1273 aa), and d) S proteins of SARS‐CoV‐2 S monomer. 3D models have been predicted by the MultiFOLD server (https://www.reading.ac.uk/bioinf/MultiFOLD/MultiFOLD_form.html) starting from the amino acids sequences. Secondary structures are represented with different colors: 𝛼‐helix in blue, 𝛽‐sheet in orange, 𝛽‐turn, and random coils in green. PyMOL was used for visualization and drawing structures.

RBD, S1, S2, and S 3D structures have been modelled with MultiFOLD software (https://www.reading.ac.uk/bioinf/MultiFOLD/MultiFOLD_form.html) for protein structure prediction, starting from their amino acids sequences.^[^ ^27^ ^]^ These sequences have been achieved by Sino Biological Europe GmbH and are reported in Supporting Information (see paragraph S1). 3D protein models are shown in **Figure** **1**. They are visualized by PyMOL and secondary structures are highlighted in blue (𝛼‐helix), orange (𝛽‐sheet), and green (𝛽‐turn and random coils).

**Figure 2:**
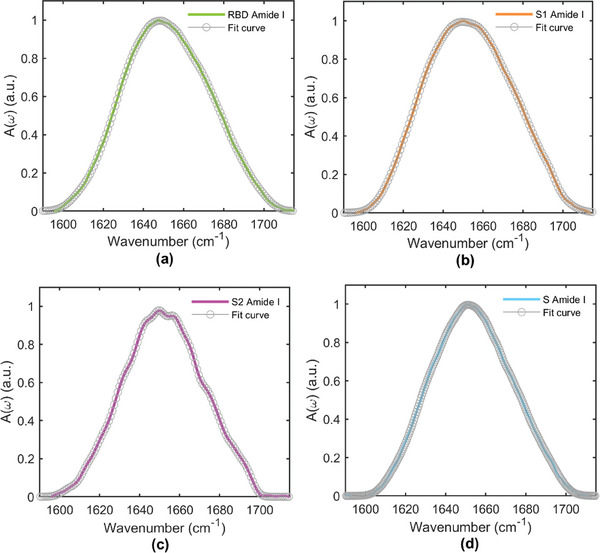
Amide I band of a) SARS‐CoV‐2 RBD, b) SARS‐CoV‐2 S1, c) SARS‐CoV‐2 S2, and d) SARS‐CoV‐2 S proteins (colored lines) and their multi‐gaussian fitting (empty grey circles).

**Figure** **2**a–d display the IR amide I absorbances A(ω) versus frequency (ω) of the SARS‐CoV‐2 RBD (green curve), SARS‐CoV‐2 S1 (orange curve), SARS‐CoV‐2 S2 (pink curve) and SARS‐CoV‐2 S (blue curve), respectively, and the total fit curves (empty grey circles), between 1590 and 1720 cm^−1^, measured at 7.4 pH and concentration of 0.25 mg mL⁻^1^.

SARS‐CoV‐2 RBD IR absorption band (Figure 2a) is centered ≈1648 cm^−1^ corresponding to the random coil vibrational mode,^[^ ^28^, ^29^ ^]^ and the Full Width at Half Maximum (FWHM) is ≈55 cm^−1^. Information on protein secondary structure of SARS‐CoV‐2 RBD has been obtained from the deconvolution of amide I bands into Gaussian spectral components through a multi‐gaussian fitting approach^[^ ^20^, ^21^, ^22^, ^27^ ^]^ (see Experimental Section). **Table** **1** summarizes the vibrational frequencies of the different fitted components, their relative percentage intensities, and their assignment to specific secondary structures.^[^ ^20^, ^21^, ^22^, ^27^, ^28^, ^29^ ^]^ According to our assignment (see paragraph 4.2), RBD amide I band shows a lower frequency peak located at 1614 cm^−1^. This contribution is associated with side chains, attributable to the IR absorption of Lys, Tyr, Asn, Trp, and Gln,^[^ ^20^, ^30^ ^]^ which are present in high percentage in its amino acids sequences, ≈24% of the overall primary sequence (see ExPASy file reported in Supporting Information). In the 𝛽‐sheet structures, the in‐phase oscillation of residues in adjacent strands gives rise to the ν⊥ 𝛽‐sheet mode^[^ ^29^, ^31^, ^32^ ^]^ with three spectral components ≈1623, 1630, and 1635 cm^−1^. Among them, the maximum absorbance of ν⊥ 𝛽‐sheet is located at 1635 cm^−1^. Instead, the in‐phase oscillation of residues in the same chain generates the ν// 𝛽‐sheet mode,^[^ ^29^, ^31^, ^32^ ^]^ located at 1689 and 1697 cm^−1^ in RBD domain. In addition, 𝛼‐helix absorbance peaks are observed at 1657 and 1662 cm^−1^; the disordered structures contribute with random coil vibrations located at 1641, 1647, and 1652 cm^−1^ and 𝛽‐turn ones at high frequencies ≈1667, 1674, and 1681 cm^−1^.

**Table 1:** Secondary structure of SARS‐CoV‐2 RBD protein in water solution as determined from the Gaussian fit analysis of amide I band.

Peak frequency [cm^−1^]	Relative area [%]	Assignment
1614	4.9	Side chain
1623	6.9	𝛽‐sheet
1630	6.3	𝛽‐sheet
1635	7.9	𝛽‐sheet
1641	10.3	Random coil
1647	12.1	Random coil
1652	8.3	Random coil
1657	7.1	𝛼‐helix
1662	7.0	𝛼‐helix
1667	9.6	𝛽‐turn
1674	8.1	𝛽‐turn
1681	6.0	𝛽‐turn
1689	3.7	𝛽‐sheet
1697	1.9	𝛽‐sheet

SARS‐CoV‐2 S1 amide I band (Figure 2b) has been deeply investigated in our previous work.^[^ ^23^ ^]^ It is centered in the random coil vibrational interval, ≈1650 cm^−1^, and its FWHM is ≈56 cm^−1^. Convoluted band frequencies, their percentage contribution and the assignments to secondary structures are obtained with the multi‐gaussian fitting approach^[^ ^20^, ^21^, ^22^, ^27^ ^]^ and are reported in our previous paper.^[^ ^23^ ^]^ Summarizing S1 results, ν⊥ 𝛽‐sheet vibration rises with two absorption peaks located at 1628 and 1633 cm^−1^, while the antiparallel ν// 𝛽‐sheet absorbs at 1693 cm^−1^. A lower frequency peak located at 1619 cm^−1^ is detected in SARS‐CoV‐2 S1 amide I band, being associated to an extended 𝛽‐sheet mode, probably involving different 𝛽‐sheet structures in the RBD region. 𝛼‐helix structures appear as a unique absorption peak located at 1659 cm^−1^. Finally, disordered structures contribute with two peaks at 1643 and 1650 cm^−1^, assignable to random coil structures, and three peaks at 1666, 1673, and 1678 cm^−1^ assignable to 𝛽‐turn.

SARS‐CoV‐2 S2 amide I band (Figure 2c) is centered ≈1651 cm^−1^ and has a FWHM of ≈48 cm^−1^. **Table** **2** lists the vibrational frequencies of the different Gaussian components obtained with the multi‐gaussian fitting approach, the relative percentage intensities and their assignments to specific secondary structures.^[^ ^20^, ^21^, ^22^, ^27^, ^28^, ^29^ ^]^ Following our assignments (see paragraph 4.2), S2 amide I band shows two peaks at low frequencies ≈1606 and 1614 cm^−1^, attributable to side chain vibrations, namely Lys, Tyr, Asn, Trp, and Gln residues, constituting ≈22% of its primary sequence (see ExPASy file in Supporting Information). The peak at 1621 cm^−1^ can be reasonably associated to an extended 𝛽‐sheet vibration, as it has been recognized in our previous spectroscopic study on monomeric S1 subunit.^[^ ^23^ ^]^ Therefore, S1 and S2 subunits both show this delocalized 𝛽‐sheet vibrations, probably involving different 𝛽‐sheet structures. On the other hand, this band seems to be not present in the S‐protein amide I band (see below, **Table** **3**). In S2, the ν⊥ 𝛽‐sheet vibration appears as a singlet ≈1631 cm^−1^, while the ν// 𝛽‐sheet vibrational mode is located at 1693 cm^−1^. A very intense absorption contribution is given by the 𝛼‐helix vibration with the two absorption peaks at 1655 and 1664 cm^−1^. They can be associated to the vibration of long and short 𝛼‐helices, respectively. Finally, random coil vibrations are located at 1639, 1644, and 1649 cm^−1^, while 𝛽‐turn are found at 1675, 1680, and 1686 cm^−1^.

**Table 2:** Secondary structure of SARS‐CoV‐2 S2 protein in water solution as determined from the Gaussian fit analysis of amide I band.

Peak frequency [cm^−1^]	Relative area [%]	Assignment
1606	1.0	Side chain
1614	1.8	Side chain
1621	5.2	𝛽‐sheet (extended)
1631	13.1	𝛽‐sheet
1639	9.8	Random coil
1644	9.5	Random coil
1649	9.1	Random coil
1655	10.3	𝛼‐helix
1664	27.3	𝛼‐helix
1675	3.4	𝛽‐turn
1680	3.7	𝛽‐turn
1686	3.5	𝛽‐turn
1692	2.4	𝛽‐sheet

**Table 3:** Secondary structure of SARS‐CoV‐2 S protein in water solution as determined from the Gaussian fit analysis of amide I band.

Peak frequency [cm^−1^]	Relative area [%]	Assignment
1615	2.3	Side chain
1625	8.2	𝛽‐sheet
1633	10.5	𝛽‐sheet
1640	8.1	𝛽‐sheet
1644	10.4	Random coil
1650	12.6	Random coil
1656	10.4	𝛼‐helix
1662	9.8	𝛼‐helix
1666	4.4	𝛽‐turn
1671	5.6	𝛽‐turn
1675	4.2	𝛽‐turn
1680	8.3	𝛽‐turn
1691	5.1	𝛽‐sheet

In SARS‐CoV‐2 S (Figure 2d), the absorption band is centered ≈1651 cm^−1^ and its FWHM is ≈50 cm^−1^. The vibrational frequencies of the different Gaussian components, their relative integrated intensities, and their assignments to specific secondary structures^[^ ^20^, ^21^, ^22^, ^27^, ^28^, ^29^ ^]^ are reported in Table 3. According to our assignment, S amide I band shows a lower frequency peak located at 1615 cm^−1^, due to the absorption of Lys, Tyr, Asn, Trp, and Gln^[^ ^20^, ^30^ ^]^ residues, constituting ≈22% of its amino acid sequence (see ExPASy file reported in Supporting Information). ν⊥ 𝛽‐sheet vibrational mode appears at frequencies of 1625, 1633, and 1640 cm^−1^, with a maximum absorbance ≈1633 cm^−1^. In addition, ν// 𝛽‐sheet mode is detected with a well‐defined absorption at 1691 cm^−1^. 𝛼‐helix absorption bands are observed at 1656 and 1662 cm^−1^. Finally, the disordered structures are recognized at 1666, 1671, 1675, and 1680 cm^−1^ for 𝛽‐turn, and at 1644 and 1650 cm^−1^ for random coil.

The area of each absorption band of the IR spectrum is assumed to be proportional to the relative amount of the secondary structure. Therefore, the relative content of each secondary structure could be estimated through the ratio among the integrated intensity of its spectral components over the total integrated intensity^[^ ^22^, ^33^ ^]^ (see Experimental Section paragraph 4.2).

**Figure 3:**
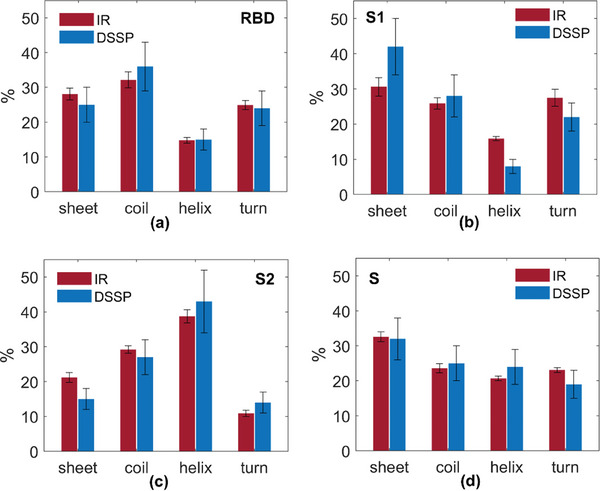
Histograms of secondary structure contents estimated by IR spectral data (red) and by MultiFOLD prediction + DSSP‐web server (blue), for RBD (a), S1 (b), S2 (c), and S (d) proteins. Errors in the experimental and modeling data are estimated as explained in Experimental Section.

The IR experimental results concerning RBD, S1, S2 and S protein secondary structures were then compared with MultiFOLD prediction models obtained starting from the amino acids sequences. DSSP‐web tool (http://bioinformatica.isa.cnr.it/SUSAN/NAR2/dsspweb.html)^[^ ^34^, ^35^ ^]^ was employed for the secondary structure assignment starting from atomic coordinates files generated with MultiFOLD.^[^ ^27^ ^]^ Both IR and DSSP results for RBD, S1, S2, and S are summarized in Tables S1–S4, (Supporting Information) and graphically displayed in **Figure** **3**a–d, respectively.

Figure 3 shows the histograms of secondary structure percentage content for RBD (a), S1 (b), S2 (c), and S (d), estimated both from IR measurements (red) and from MultiFOLD+DSSP prediction (blue). Experimental errors are calculated as explained in Experimental Section, while errors for DSSP are assumed to be 20% of the value (https://scratch.proteomics.ics.uci.edu/explanation.html#SSpro8). Inspection of Figure 3 suggests a good agreement between the secondary structure contents estimated via IR spectroscopy and via MultiFOLD + DSSP modeling.

RBD protein structure is mostly constituted of disordered structures (32.2% of random coil and 24.9% of 𝛽‐turn). 𝛽‐sheet percentage content results to be 28.1%, while the minor content is given by 𝛼‐helix (14.8%) (see Figure 3a). Referring to our past work,^[^ ^23^ ^]^ S1 protein shows a 𝛽‐sheet percentage content of 30.6% and an 𝛼‐helix content of 15.9%, while disorder structures contribute with 25.9% of random coil and 27.5% of 𝛽‐turn (see Figure 3b). S2 is the domain giving the strongest contribution of 𝛼‐helix structures, constituting 38.7% of the protein; 𝛽‐sheet percentage content results to be 21.2%, while random coil and 𝛽‐turn constitute the 29.2% and 10.9%, respectively (see Figure 2c). Finally, S protein structure is overall constituted by an 𝛼‐helix content of 20.7% and a 𝛽‐sheet content of 32.6%, while disordered structures contribute with random coils (23.6%) and 𝛽‐turn (23.1%) (see Figure 3d).

## 3 Discussion

In this work, we investigated RBD, S1, S2, and S proteins from SARS‐CoV‐2 virus in terms of their secondary structure content, conformational order and interaction with the solvent, via ATR‐IR spectroscopy by measuring their amide I vibrational bands (1580–1720 cm^−1^) at serological pH (7.4). The advantage of vibrational spectroscopy, in particular IR spectroscopy, lies in the ability to study proteins in close proximity to the physiological environment and/or in vivo, simultaneously obtaining information on protein dynamics, hydrophobicity, and structural order, differently from other techniques. Concerning the protein secondary structure, certain structures may be crucial for binding to other molecules, for the catalysis of chemical reactions, or for the folding and assembly processes of proteins themselves. Therefore, understanding the secondary structure of a protein provides an essential basis for studying and understanding its biological role and interactions and potentially developing targeted actions, designing drugs or biosensors and biomedical nanostructures.^[^ ^36^, ^37^, ^38^, ^39^, ^40^ ^]^ The investigation of the secondary structure of S protein is a key point for understanding its structural behavior, affecting its functionalities. S protein is the anchoring site between the virus and the host during the infectious process, through the interaction between the receptor and the RBD region, while S1 and S2 are the two subunits constituting its overall structure. With the IR spectroscopy analysis, we aim to follow the structural conformation characterizing the different regions of S protein, starting from the simpler element of RBD, through S1^[^ ^23^ ^]^ and the S2 subunits, up to the whole S protein, interpreting the spectroscopic results and observing how these features change as long as the amino acids sequence grows up until the configuration of the final S protein is attained. Proteins secondary structure and 3D conformation define what is their functionality. Therefore, the spectral analysis and the structural investigation of RBD, S1, S2, and S proteins in terms of their respective secondary structure, hydrophilicity, and conformational order, can in principle provide a complementary description to what it is known about their different roles. During the infectious process, RBD is subjected to down‐up movement and it directly forms the chemical bond with ACE2, while from spectral analysis it results to be the most hydrophilic and disordered domain of S protein. NTD, together with RBD, constitutes the S1 subunit. It is known to play a role in the recognition of some protein receptors and/or in the prefusion‐to‐postfusion transition.^[^ ^41^, ^42^ ^]^ It provides an overall more hydrophobic and ordered conformation to the S1 subunit. Finally, S2 subunit undergoes a conformational rearrangement mediating the fusion with ACE2 and it resulted to be characterized by a hydrophobic and ordered structure.

The inspection of the IR absorption band of RBD protein (319–541 aa, see Figure 2a) shows that its secondary structure is mostly constituted ≈57% of disordered domains (32.2% of random coil and 24.9% of 𝛽‐turn). As already well known from previous works,^[^ ^20^, ^32^, ^43^, ^44^ ^]^ more disordered structures are associated with a broader IR absorption band (for RBD, the amide I FWHM ≈55 cm^−1^). On the other hand, more ordered conformations (where order is intended as secondary structure symmetry in terms of bonds and angles) give rise to a larger delocalization of the vibrational mode and, therefore, to a sharper absorption peak. The extent of conformational order is deduced comparing the IR results with the DSSP secondary structure assignment.^[^ ^34^, ^35^ ^]^ Indeed, analyzing DSSP prediction, in RBD protein we observe the presence of only short 𝛼‐helices, i.e., less than 10 aa (14.8%, Figure S2, Supporting Information) and 𝛽‐sheets with few numbers of ladders and short chains (28.1%, Figures S3–S5, Supporting Information), suggesting an overall poorly structured conformation. On the other hand, we cannot exclude the contribution to structural disorder also of other parameters, such as the interaction with the solvent.^[^ ^32^, ^44^, ^48^ ^]^ The bonding among water molecules and protein amino acids contributes to a partial unfolding of local structures and therefore to the broadening of the amide I band. Gravy value was calculated (see ExPASy file in SI) and, in this case, it results to be −0.376, revealing a hydrophilic behavior of RBD domain. Thus, we can conclude that the broadening of the amide I band is due to the competitive action of both an intrinsic and an externally induced structural disorder. The hydrophilic behavior of RBD protein can be also recognized by the position of the absorption maximum, located at 1648 cm^−1^, therefore slightly shifted to lower frequency compared to S1, S2, and S protein (see below), as H‐bonds among amino acids and water molecules are supposed to influence the amide group vibration, decreasing its overall oscillation frequency^[^ ^29^, ^49^, ^50^ ^]^ and leading to the amide I band redshift.

Moving first to S1 protein, it is the subunit (1‐685 aa) containing both the RBD domain (previously discussed) and other protein regions rich of 𝛽‐sheets and disordered structures (see Figure 1b). From Figure 2b, the S1 amide I band maximum is located ≈1650 cm^−1^, slightly higher in frequencies with respect to the RBD amide I domain. Indeed, S1 Gravy value is −0.256 (see ExPASy file in Supporting Information) pointing to a slightly more hydrophobic behavior if compared to the simpler RBD domain. S1 amide I band is still remarkably broad (FWHM is 56 cm^−1^) even if the protein shows the presence of highly ordered 𝛽‐sheet structures, with a high number of ladders and parallel and antiparallel bridges (see Figures S3–S5, Supporting Information). On the other hand, it contains only few short 𝛼‐helices (15.9%) (see Figure S2, Supporting Information), whose signal arises as a singlet located at 1659 cm^−1^. S1 protein shows a strong contribution of 𝛽‐sheet structures (30.6%), in combination with a low content of 𝛼‐helices (15.9%). Finally, as already reported in our previous work,^[^ ^23^ ^]^ it is worth underlining the presence in S1 amide I band of a low frequency peak at 1619 cm^−1^, which has been attributed to a delocalized vibration, probably involving different 𝛽‐sheet structures.

S2 protein is the other subunit constituting the overall S protein (686‐1273 aa) (Figure 1c). The maximum of its amide I band is located ≈1651 cm^−1^, slightly blue shifted with respect to S1, showing a significantly narrower shape (FWHM 48 cm^−1^). From DSSP, S2 protein results to have few 𝛽‐sheets structures, characterized by both small and large number of bridges and only with few numbers of ladders (see Figure S3 and S4, Supporting Information), but it also shows the presence of very long 𝛼‐helices, up to 30 aa, giving a notably structural order to the protein. Moreover, S2 protein Gravy value is −0.118, meaning it has an even stronger hydrophobic behavior with respect to RBD and S1 domains. These two factors contribute to the sharp S2 amide I band. S2 shows a very strong contribution of 𝛼‐helices (38.7%), differently from RBD and S1, corresponding to two intense absorptions at 1655 and 1664 cm^−1^, respectively. The first one is assignable to long 𝛼‐helices (>30 aa) (see Figure S2, Supporting Information), while the band located at higher frequencies is attributable to short 𝛼‐helices.^[^ ^45^ ^,^ ^46^ ^]^ S2 amide I band shows only a peak in the low frequency interval attributable to ν⊥ 𝛽‐sheet vibration (specifically, at 1631 cm^−1^). Indeed, S2 is the domain providing the minor contribution of 𝛽‐sheet structures (only 21.2%) to the overall S protein. ν// 𝛽‐sheet vibration appears as a single absorption peak located at 1693 cm^−1^. S2 amide I band also shows the presence of a low frequency peak at 1621 cm^−1^ which, in accordance with our previous work on S1 subunit, can be attributed to an extended 𝛽‐sheet vibration, possibly delocalized along different sheets of S2 protein.

S1 and S2 domains together constitute the S monomeric protein (Figure 1d). As expected, assembling multidomain protein structures results in new self‐stabilizing folding,^[^ ^51^ ^]^ with new chemical interactions that are not the simple sum of S1 and S2 ones (see Figures S2–S5, Supporting Information). Therefore, S protein amide I band is not a trivial combination of S1 and S2 secondary structures spectral components. On the other hand, as already discussed, S protein spectral features are influenced by its functionalities, such as the overall hydrophilicity and the structural order, and these ones are on their side the result of S1 and S2 functionalities combination. Indeed, S protein amide I band evidently shows a shape with an absorption maximum located at 1651 cm^−1^ and a FWHM of 50 cm^−1^ (see Figure 2d). In accordance, its Gravy value results to be −0.177, and DSSP prediction shows the presence of long 𝛼‐helices (Figure S2, Supporting Information) and highly structured 𝛽‐sheets (Figures S3–S5, Supporting Information), providing a great extent of conformational order. Actually, we can attribute the more hydrophobic behavior to the transmembrane region (S2 protein), made up of a bundle of hydrophobic long α‐helices. Finally, the overall S protein results to be characterized by the presence of 𝛽‐sheet structures constituting ≈32% of the structure and both long and short 𝛼‐helices, which appear with two absorption peaks at 1656 and 1662 cm^−1^, respectively, constituting about the 21% of the whole structure.

## 4 Conclusion

In this work we report for the first time, at the best of our knowledge, on IR vibrational spectroscopic study of SARS‐CoV‐2 S glycoprotein monomer and of its constituting domains, i.e., RBD, S1 and S2 components. We studied the RBD domain which anchors the whole protein to the host receptor ACE2, the S1 region hosting the RBD domain, the S2 subunit, and the whole S protein at serological pH (7.4), interpreting the results in terms of their secondary structure, hydrophobicity, and conformational order.

Focusing on the amide I vibrational band (1590–1720 cm^−1^), we estimated the secondary structure percentage contents (in terms of 𝛽‐sheet, random coil, 𝛼‐helix and 𝛽‐turn contents) for RBD, S1, S2, and S proteins. The experimental secondary structure contents are compared with DSSP assignments of MultiFOLD predicted models for all four proteins, finding a good agreement between experiments and models. Variations in proteins spectral features and secondary structure have been recognized as long as we move from the simpler RBD domain, to the more complex S1 and S2 subunits, up to the whole S protein. The blueshift of amide I absorption maximum and the narrowing of the band can be attributed to the combined effect of the increase of intrinsic structural order and the increase of proteins' hydrophobic behavior. The comparison between the experimental data and the DSSP assignment on predicted models allow us to interpret the spectral results in terms of the extent of order in 𝛼‐helices and 𝛽‐sheets structures, referring to the separate contribution of long and short 𝛼‐helices and of more or less structured 𝛽‐sheets.

In conclusion, these results confirm the excellent capability of IR spectroscopy to provide rapid and insightful information on protein secondary structures, shedding light on various aspects, such as the hydrophobicity, the conformational order and functionalities, from each protein domain to complex S structure. The knowledge of the secondary structural characteristics of SARS‐CoV‐2 S protein and its components is of primary importance for understanding the mechanisms occurring in the viral process and to address specific actions aimed at the development of specific drugs, preventing actions and diagnostic tools, such as the design of optical biosensors.

## 5 Experimental Section

### Protein Preparation

Recombinant S1+S2 ECD (Cat. 40589‐V08B1, aa 1209, purity > 90%), S1 (Cat. 40591‐V08B1, aa 681, purity > 90%), S2 ECD (Cat. 40590‐V08B, aa 539, purity >90%), and RBD (Cat. 40592‐V08B, aa. 234, purity >95%) proteins monomers, fused with a polyhistidine tag (His tag) at the C terminus SARS‐CoV‐2 were purchased from Sino Biological Europe GmbH (Eschborn, Germany).

They were expressed in baculovirus insect cells, with the purity (>90%) determined by sodium dodecyl sulphate–polyacrylamide gel electrophoresis (SDS‐PAGE), and finally used without further purification.

The study was carried out on a dataset of SARS‐CoV‐2 proteins collected in late spring 2020, and it was referred to the alpha variant that affected Europe and Italy in the pandemic crisis of March 2020. The lyophilized proteins were reconstructed by dissolving 100 µg in distilled water (400 µL) at pH 7.4 (0.25 mg mL⁻^1^ concentration).

### Attenuated‐Total‐Reflection Infrared Spectroscopy and Data Analysis

ATR‐IR spectra of the recombinant protein monomers of SARS‐CoV‐2 glycoproteins were collected using a Bruker (Billerica, MA, USA) Vertex 70v Michelson spectrometer equipped with an ATR–Diamond single reflection module and a DLaTGS wide range detector. Spectroscopic measurements were carried out at room temperature (26 °C) and under vacuum conditions to mitigate the interferences induced by water vapor and CO_2_ absorptions. The background spectrum (aqueous solution) was recorded immediately prior to each sample measurement. A drop of five microliters of the sample solutions was placed directly on the diamond crystal, and 128 scans between 400 and 4000 cm^−1^ with a resolution of 2 cm^−1^ were acquired. Each ATR spectrum results from the average of five independent measurements. The ATR crystal was cleaned with ethanol (purity > 90%), distilled water and subsequently with a lens tissue in order to eliminate any spurious signal. Sample solutions were measured at different concentrations, successfully verifying that the IR measurement was not dependent on the concentration. Raw data were visualized and analyzed using OPUS 8.2. software (Bruker Optics) and algorithms based on MATLAB (ver. 2018, MathWorks Inc., Natick, MA, USA). To obtain the protein absorption spectra A(ω), we subtracted the spectrum of the aqueous solution (see [23]) to eliminate the contribution of the background^[^ ^29^, ^52^ ^]^ and applied the ATR correction algorithm and a piecewise linear baseline subtraction. The secondary structures of the monomeric units of SARS‐CoV‐2 glycoprotein were obtained by the decomposition of the amide I vibrational absorption band^[^ ^22^, ^28^ ^]^ into its spectral components. The amide I band was deconvoluted with 2^nd^ derivative analysis and a multicomponent Gaussian fitting.

In particular, the frequencies, achieved by 2^nd^‐derivative spectra, were used as starting conditions for Gaussian curve fitting, and the residual error (RMSE) was employed for assessing the convolution procedure performance.

Assuming that any protein can be considered as a linear sum of a few fundamental secondary structural elements, the intensity of each component peak, normalized to the total intensity, was used to calculate the percentage of each absorption band and then to estimate the secondary structures of the protein units.^[^ ^8^, ^22^, ^33^, ^47^ ^]^ In this specific case, there was a strong contribution of the amino acid side chain (≈1615 cm^−1^) in this spectral region, that were removed in the integrated intensity evaluations.^[^ ^53^ ^]^ The error associated with each secondary structure percentage content is calculated propagating the standard deviations of the convoluted band integrals percentage contribution obtained for each protein measurement run by adapting the final fit on its spectrum.

### MultiFOLD and DSSP Prediction

MultiFOLD server (https://www.reading.ac.uk/bioinf/MultiFOLD/MultiFOLD_form.html) was employed for protein predictions, in order to obtain accurate models of S, S1, and RBD proteins tertiary structures, starting from their experimental amino acid sequences.^[^ ^27^ ^]^ The MultiFOLD routine is based on three stages (modeling, scoring, and refinement), which allows the user to obtain the five most accurate generated models. Predicted local distance difference test (plDDT)^[^ ^54^ ^]^ and the template modeling (TM) score^[^ ^55^ ^]^ were used to evaluate the best protein 3D model.

Atomic files obtained with MultiFOLD have then been analyzed with DSSP server for secondary structure assignments, based on the analysis of backbone dihedral angles and hydrogen bonds (http://bioinformatica.isa.cnr.it/SUSAN/NAR2/dsspweb.html).^[^ ^34^, ^35^ ^]^

## Conflict of Interest

The authors declare no conflict of interest.

## Supporting information

Supporting Information

## Data Availability

The data that support the findings of this study are available from the corresponding author upon reasonable request.
