# Supplementary material for: Infrared Spectroscopy of SARS‐CoV‐2 Viral Protein: from Receptor Binding Domain to Spike Protein
Source: Adv Sci (Weinh). 2024 Jul 12;11(39):2400823. doi: 10.1002/advs.202400823 (PMC11497030; doi:10.1002/advs.202400823)
Supplement: Supplementary file 1 — Supporting Information [file ADVS-11-2400823-s001.pdf]

ADVANCED SCIENCE
Open Access

Supporting Information

for *Adv. Sci.*, DOI 10.1002/advs.202400823

Infrared Spectroscopy of SARS-CoV-2 Viral Protein: from Receptor Binding Domain to Spike Protein

Tiziana Mancini, Salvatore Macis, Rosanna Mosetti, Nicole Luchetti, Velia Minicozzi, Andrea Notargiacomo, Marialilia Pea, Augusto Marcelli, Giancarlo Della Ventura, Stefano Lupi and Annalisa D'Arco**

Supporting Information

Title: Infrared Spectroscopy of SARS-CoV-2 Viral Protein: from Receptor Binding Domain to Spike Protein

Tiziana Mancini, Salvatore Macis, Rosanna Mosetti; Nicole Luchetti, Velia Minicozzi, Andrea Notargiacomo, Marialilia Pea, Augusto Marcelli, Giancarlo Della Ventura, Stefano Lupi and Annalisa D'Arco**

Correspondence

Tiziana Mancini, Annalisa D'Arco

Department of Physics, University La Sapienza, P.le A. Moro 2, 00185, Rome, Italy

E-mail: tiziana.mancini@uniroma1.it, annalisa.darco@uniroma1.it

S1. Amino acids sequences

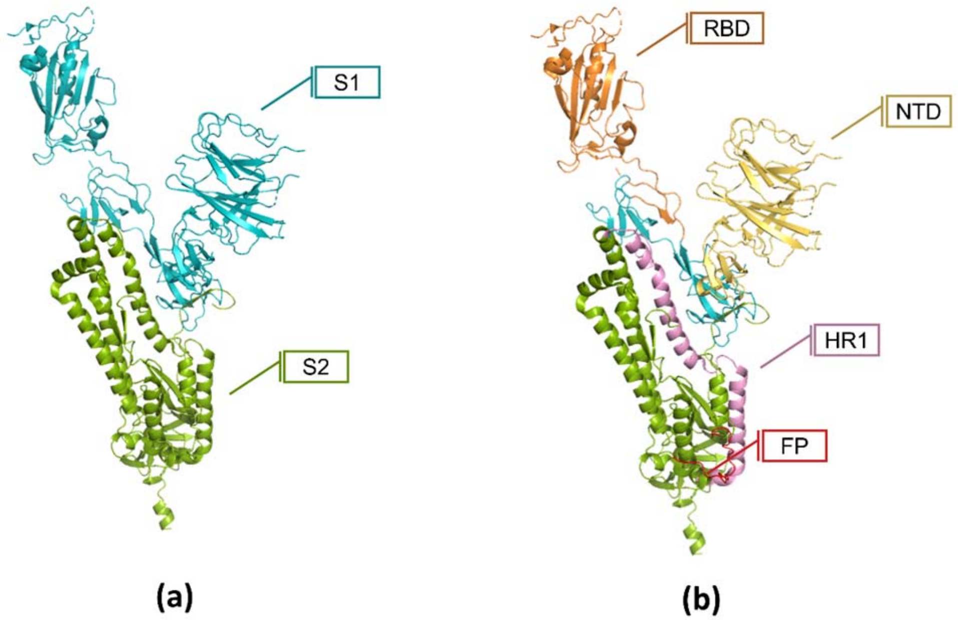

Figure S1. The structure of chain A of SARS-CoV-2 spike protein, taken from Protein Data Bank (ID: 6vsb) by Wrapp et al. [6]. We report (a) S1 (cyan, 1–685 aa), and S2 (green, 686–1,273 aa) subunits, respectively. (b) S1 subunit includes NTD domain (yellow, 14–305 aa) and RBD (orange, 319–541 aa), and S2 subunit includes FP (red, 788–806 aa), hepta-peptide repeat sequence 1 HR1 (pink, 912-984 aa). PyMOL was used for visualization and drawing structure.

SARS-CoV-2 RBD protein amino acids sequence

RVQPTESIVRFPNITNLCPFGEVFNATRFASVYAWNRKRISNCVADYSVLYNSASFSTFKCYGVSPTKLNDLCFTN VYAD
SFVIRGDEVRQIAPGQTGKIADYNYKLPDDFTGCVIAWNSNNLDSKVGGNYNYLYRLFRKSNLKPFERDISTEIY QAGST
PCNGVEGFNCYFPLQSYGFQPTNGVGYQPYRVVVLSFELLHAPATVCGPKKSTNLVKNKCVNFAHHHHHHHHHH

SARS-CoV-2 S1 protein amino acids sequence

VNLTTRTQLPPAYTNSFTRGVYYPDKVFRSSVLHSTQDLFLPFFSNVTWFHAIHVSGTNGTKRFDNPVLPFNDGVYFASTEKS
NIIRGWIFGTTLDSKTQSLLIVNNATNVVIKVCEFQFCNDPFLGVYYHKNNKSWMESEFRVYSSANNCTFEYVSQPFLMDLE
GKQGNFKNLREFVFKNIDGYFKIYSKHTPINLVRDLPQGFSALEPLVDLPIGINITRFQTLLALHRSYLTPGDSSSGWTAGAAAY
YVGYLQPRTFLLKYNENGTITDAVDCALDPLSETKCTLKSFTVEKGIYQTSNFRVQPTESIVRFPNITNLCPFGEVFNATRFASVY
AWNRKRISNCVADYSVLYNSASFSTFKCYGVSPTKLNDLCFTNVYADSFVIRGDEVRQIAPGQTGKIADYNYKLPDDFTGCVIA
WNSNNLDSKVGGNYNYLYRLFRKSNLKPFERDISTEIYQAGSTPCNGVEGFNCYFPLQSYGFQPTNGVGYQPYRVVVLSFEL
LHAPATVCGPKKSTNLVKNKCVNFNFNGLTGTGVLTESNKKFLPFQQFGRDIADTTDAVRDPQTLEILDITPCSFGGVSVITPG
TNTSNQVAVLYQDVNCTEVPVAIHADQLTPTWRVYSTGSNVFQTRAGCLIGAEHVNNSYECDIPIGAGICASYQTQTNSPRR
ARAHHHHHHHHHH

SARS-CoV-2 S2 protein amino acids sequence

SVASQSIIAYTMSLGAENSVAYSNNSIAIPTNFTISVTTEILPVSMTKTSVDCTMYICGDSTECSNLLLQYGSFCTQL
NRALTGIAVEQDKNTQEVFAQVKQIYKTPPIKDFGGFNFSQILPDPSKPSKRSFIEDLLFNKVTLADAGFIKQYGD
CLGDIAARDLICAQKFNGLTVLPPLLTDEMIAQYTSALLAGTITSGWTFGAGAALQIPFAMQMAYRFNGIGVTQ
NVLYENQKLIANQFNSAIGKIQDSLSSTASALGKLQDVVNQNAQALNTLVKQLSSNFGAISSVLNDILSRLDKVEA
EVQIDRLITGRLQSLQTYVTQQLIRAAEIRASANLAATKMSECVLGQSKRVDFCGKGYHLMSFPQSAPHGVVFL
HVTYVPAQEKNFTTAPAICHDGKAHFPREGVFVSNGTHWFVTQRNFYEPQIITTDNTFVSGNCDVVIGIVNNT
VYDPLQPELDSFKEELDKYFKNHTSPDVDLGDISGINASVVNIQKEIDRLNEVAKNLNESLIDLQELGKYEQYIKW
PAHHHHHHHHHH

SARS-CoV-2 S protein amino acid sequence

VNLTTRTQLPPAYTNSFTRGVYYPDKVFRSSVLHSTQDLFLPFFSNVTWFHAIHVSGTNGTKRFDNPVLPFNDGVYFASTEKS
NIIRGWIFGTTLDSKTQSLLIVNNATNVVIKVCEFQFCNDPFLGVYYHKNNKSWMESEFRVYSSANNCTFEYVSQPFLMDLE
GKQGNFKNLREFVFKNIDGYFKIYSKHTPINLVRDLPQGFSALEPLVDLPIGINITRFQTLLALHRSYLTPGDSSSGWTAGAAAY
YVGYLQPRTFLLKYNENGTITDAVDCALDPLSETKCTLKSFTVEKGIYQTSNFRVQPTESIVRFPNITNLCPFGEVFNATRFASVY
AWNRKRISNCVADYSVLYNSASFSTFKCYGVSPTKLNDLCFTNVYADSFVIRGDEVRQIAPGQTGKIADYNYKLPDDFTGCVIA
WNSNNLDSKVGGNYNYLYRLFRKSNLKPFERDISTEIYQAGSTPCNGVEGFNCYFPLQSYGFQPTNGVGYQPYRVVVLSFEL
LHAPATVCGPKKSTNLVKNKCVNFNFNGLTGTGVLTESNKKFLPFQQFGRDIADTTDAVRDPQTLEILDITPCSFGGVSVITPG
TNTSNQVAVLYQDVNCTEVPVAIHADQLTPTWRVYSTGSNVFQTRAGCLIGAEHVNNSYECDIPIGAGICASYQTQTNSPRR
ARSVASQSIIAYTMSLGAENSVAYSNNSIAIPTNFTISVTTEILPVSMTKTSVDCTMYICGDSTECSNLLLQYGSFCTQLNRALTG
IAVEQDKNTQEVFAQVKQIYKTPPIKDFGGFNFSQILPDPSKPSKRSFIEDLLFNKVTLADAGFIKQYGDCLGDIAARDLICAQK
FNGLTVLPPLLTDEMIAQYTSALLAGTITSGWTFGAGAALQIPFAMQMAYRFNGIGVTQNVLYENQKLIANQFNSAIGKIQD
SLSSTASALGKLQDVVNQNAQALNTLVKQLSSNFGAISSVLNDILSRLDKVEAEVQIDRLITGRLQSLQTYVTQQLIRAAEIRAS
ANLAATKMSECVLGQSKRVDFCGKGYHLMSFPQSAPHGVVFLHVTYVPAQEKNFTTAPAICHDGKAHFPREGVFVSNGTH
WFVTQRNFYEPQIITTDNTFVSGNCDVVIGIVNNTVYDPLQPELDSFKEELDKYFKNHTSPDVDLGDISGINASVVNIQKEID
RLNEVAKNLNESLIDLQELGKYEQYIKWPAHHHHHHHHHH

S2. Secondary structure percentage content estimation from IR measurements and MultiFOLD+DSSP prediction

Table S1. Comparison among RBD protein secondary structure percentage contents estimated by the Gaussian decomposition of the vibrational absorption spectrum and MultiFOLD+DSSP calculation.

	IR (%)	DSSP (%)
β-sheet	28.1 ± 1.7	25 ± 5
Random coils	32.2 ± 2.3	36 ± 7
α-helix	14.8 ± 0.8	15 ± 3
β-turn	24.9 ± 1.3	24 ± 5

Table S2. Comparison among S1 protein secondary structure percentage contents estimated by the Gaussian decomposition of the vibrational absorption spectrum and MultiFOLD+DSSP calculation.

	IR (%)	DSSP (%)
β-sheet	30.6 ± 2.6	42 ± 8
Random coils	25.9 ± 1.6	28 ± 6
α-helix	15.9 ± 0.6	8 ± 2
β-turn	27.5 ± 2.4	22 ± 4

Table S3. Comparison among S2 protein secondary structure percentage contents estimated by the Gaussian decomposition of the vibrational absorption spectrum and MultiFOLD+DSSP calculation.

	IR (%)	DSSP (%)
β-sheet	21.2 ± 1.4	15 ± 3
Random coils	29.2 ± 1.1	27 ± 5
α-helix	38.7 ± 1.9	43 ± 9
β-turn	10.9 ± 0.9	14 ± 3

Table S4. Comparison among S protein secondary structure percentage contents estimated by the Gaussian decomposition of the vibrational absorption spectrum and MultiFOLD+DSSP calculation.

	IR (%)	DSSP (%)
β-sheet	32.6 ± 1.4	32 ± 6
Random coils	23.6 ± 1.3	25 ± 5
α-helix	20.7 ± 0.7	24 ± 5
β-turn	23.1 ± 0.7	19 ± 4

S3. DSSP server secondary structure prediction

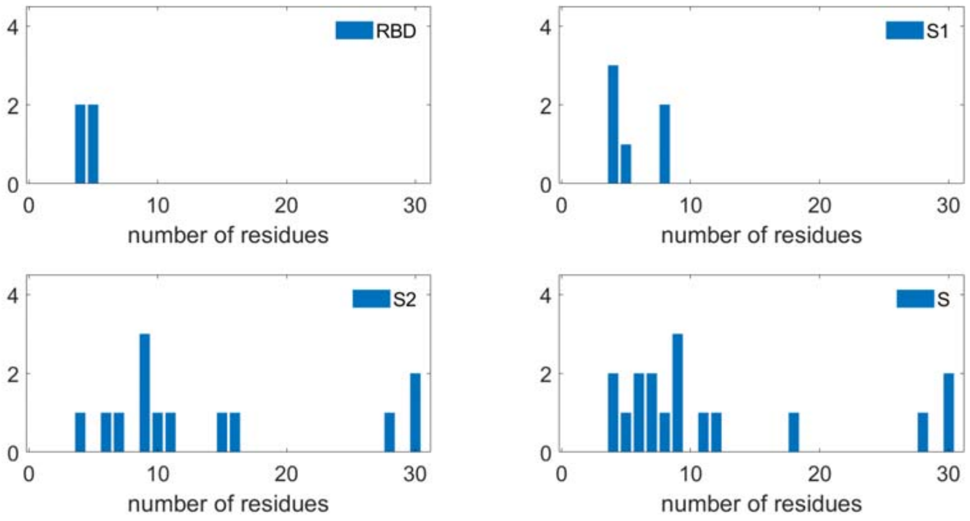

Fig S2. Histograms of number of α-helices having a certain number of residues in (a) S protein, (b) S1 protein and (c) RBD protein.

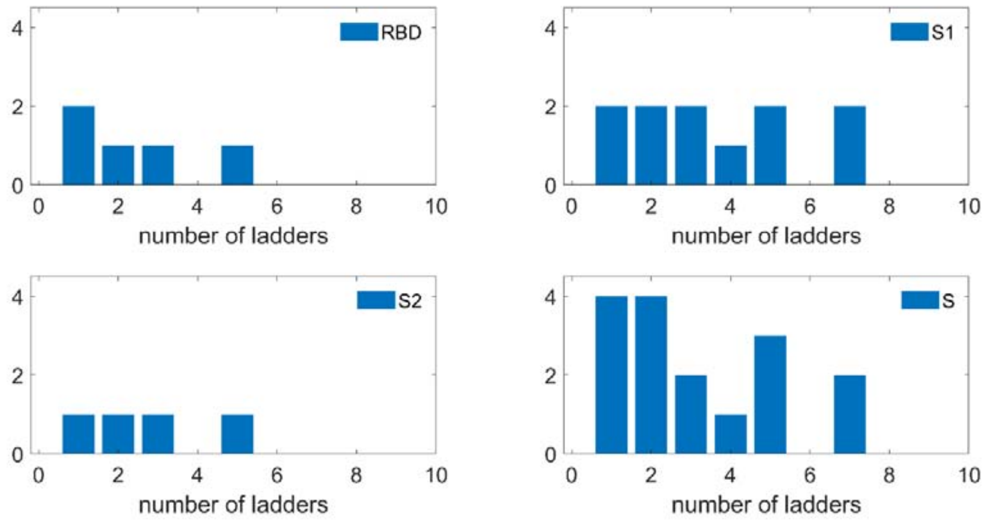

Fig S3. Histograms of number of β-sheet structures having a certain number of ladders in (a) S protein, (b) S1 protein and (c) RBD protein.

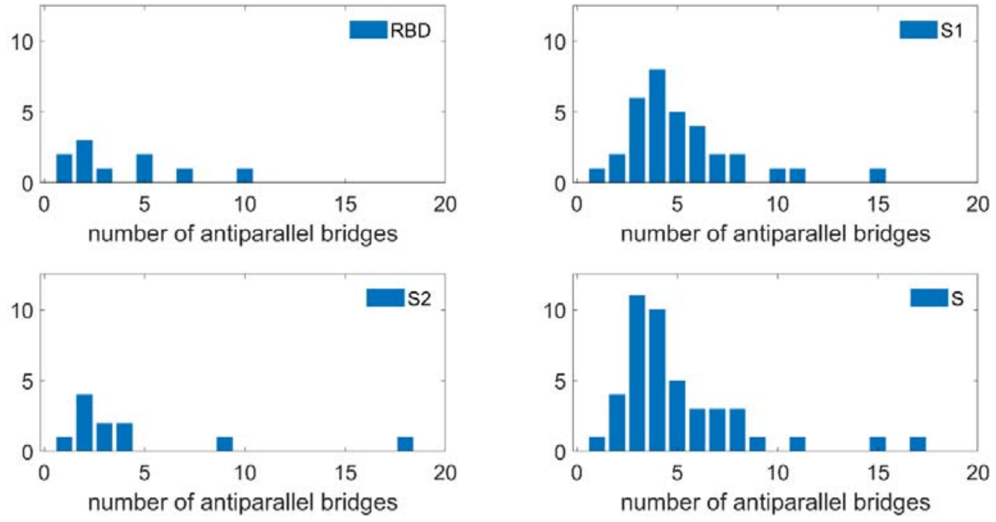

Fig S4. Histograms of number of strands having a certain number of antiparallel bridges in (a) S protein, (b) S1 protein and (c) RBD protein.

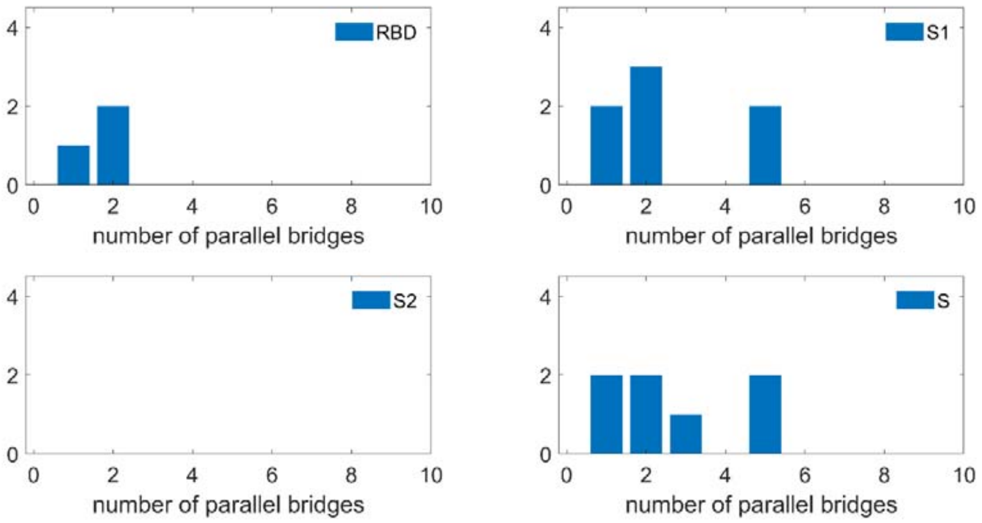

Fig S5. Histograms of number of strands having a certain number of parallel bridges in (a) S protein, (b) S1 protein and (c) RBD protein.
